# Immobilization of laccase onto modified PU/RC nanofiber via atom transfer radical polymerization method and application in removal of bisphenol A

**DOI:** 10.1002/elsc.201900075

**Published:** 2019-11-04

**Authors:** Xin Li, Dawei Li, Pengfei Lv, Jinyan Hu, Quan Feng, Qufu Wei

**Affiliations:** ^1^ Key Laboratory of Eco‐Textiles, Ministry of Education Jiangnan University Jiangsu Province Wuxi P. R. China; ^2^ Fujian Key Laboratory of Novel Functional Textile Fiber and Materials Minjiang University Fuzhou Fujian P. R. China; ^3^ Key Laboratory of Textile Fabric Anhui Polytechnic University Wuhu Anhui P. R. China

**Keywords:** ATRP, bisphenol A, Laccase immobilization, nanofiber

## Abstract

In this study, 2‐hydroxyethyl methacrylate (HEMA) was used as the monomers for surface grafting on electrospun PU/RC nanofiber membrane via atom transfer radical polymerization (ATRP) method, and the PU/RC‐poly(HEMA) nanofiber membrane was investigated as a carrier for LAC. Free and immobilized LAC was characterized, and efficiency of bisphenol A (BPA) removal was determined. The results indicated that the PU/RC‐poly(HEMA)‐LAC showed relatively higher pH stability, temperature stability, and storage stability than free and PU/RC‐LAC; moreover, more than 60% of the PU/RC‐poly(HEMA)‐LAC activity was retained after 10 cycles of ABTS treatment. Notably, the BPA removal efficiency of PU/RC‐poly(HEMA)‐LAC membrane generally ranged from 87.3 to 75.4% for the five cycles. Therefore, the PU/RC‐poly(HEMA) nanofiber membrane has great potential as a carrier for the LAC immobilization for various industrial applications and bioremediation.

**Abbreviations**
ABTS: 2,20‐azinobis‐(3‐ethylbenzothiazoline‐6‐sulfonic acid)
ATRP: atom transfer radical polymerization
BPA: bisphenol A
CA: cellulose acetate
HEMA: 2‐hydroxyethyl methacrylate
HMTETA: 1,1,4,7,10,10‐hexamethyltriethyl‐enetetramine
LAC: laccase
PU: polyurethane
TEA: triethylamine

## 1 INTRODUCTION

Wastewater discarded from processing industries 1, contains dissolved organic pollutants such as phenols and substituted phenolic compounds 2, which are toxic and hazardous to the environment 3. Among these compounds, bisphenol A (BPA) (2,2‐bis (4‐hydroxyphenyl) propane), which is widely used in the production of plastics 4, has been identified as an endocrine disrupting chemicals by the US Environmental Protection Agency (EPA) and the World Wildlife Fund for Nature (WWF) and has been declared as a social, environmental and global issue 5. This has made BPA contamination in the environment of a great worldwide concern 6, hence, methods to efficiently remove BPA from the environment are urgently required 7.

In recent years, enzymes have attracted special attention, due to its high efficiency and superior substrate selectivity 8. The ability of enzymes to oxidize a wide range of organic compounds has great potential in wastewater treatment 9, bioremediation 10, and production of polymers with special properties 11. Laccase (LAC) is a multi‐copper oxidase which has been found and produced by Trametes fungi, with effectively catalyzing the oxidation/decomposition of various organic pollutants 12. However, the application of free LAC is hampered by several problems 13. LAC, for example, is sensitive to the variations of temperature and pH value 14. Therefore, it is easy to lose its catalytic activity in hazardous environment 15. In addition, free enzymes are relatively difficult to be separated from the product, resulting in secondary pollution 16. Immobilization of enzymes is an effective way to solve these problems 17. A critical challenge for enzyme immobilization is to develop high‐performance carriers 18. The characteristics of an ideal carrier for enzyme immobilization include inexpensive nature 19, being able to load a significant amount of enzyme per unit weight 20, low hydrophobicity 21, inertness after immobilization 22, microbial resistance 23, pH, thermal, and mechanical resistance 24. Conventionally, nanoparticles and microspheres have been used as carriers for immobilized enzymes because of their large specific surface area and porosity, resulting in higher enzyme loading of the immobilization process 25.

Presently, electrospun nanofiber membrane with high specific surface area and porosity 26, good structure and characteristics of modified 27, are considered to have good application prospects in the field of immobilized enzymes 28. One polymer that has been electrospun into nanofiber membrane for LAC immobilization is cellulose acetate (CA), which can be used in produce regenerated cellulose (RC) nanofiber membrane while undergoes hydrolysis in NaOH aqueous solution upon simply immersed at room temperature 29. The repeating unit of cellulose macromolecule has three hydroxyl (‐OH) groups, which can be utilized to immobilize enzyme 30, in addition, cellulose can be chemically modified to enhance the immobilized performance/capability. Another block copolymer of polyurethane (PU) was selected due to its superior flexibility and elasticity 31.

It is worth noting that the surface of electrospun nanofiber membrane exist a certain number of chemical/physical active sites for enzymes immobilization, but not enough 32. Atom transfer radical polymerization (ATRP) was adopted in this research, in order to introduce more active sites on the surface of nanofiber membrane, so as to increase the amount of enzyme per unit weight 33. ATRP was discovered in 1995 in conjunction with that Sawamoto and Matyjaszewski exploited the redox chemistry of ruthenium and copper complexes 34, respectively, and rapidly become one of the most versatile reversible deactivation radical polymerization methods due to the simple experimental setup, broad range of monomers and solvent used, commercial availability of initiators (alkyl halides) and catalyst components 35, and also recognizes opportunities for further modification by easy attachment of initiators to surfaces or biological molecules 36. The ATRP method has often been adopted for making linear polymer chains/brushes with controlled molecular lengths/weights since the propagation centers do not undergo chain termination and/or chain transfer during polymerization 37, and thus the molecular weights/lengths increase linearly with the conversion of monomers 38, and the monomers involved in ATRP are very various 39, for example, styrene 40, acrylonitrile 41, acrylate 42, acrylamide 43, and so on.

In this study, the electrospun PU/RC‐poly(HEMA) blend nanofiber membrane was first prepared and then used as an ideal carrier for LAC immobilization by ion coordination method. This study explored various parameters related to LAC immobilization including the amount, temperature and pH stability, storage stability and reusability, and efficiency of BPA removal. The study demonstrated that PU/RC‐poly(HEMA) has great potential as a carrier for the LAC immobilization for various industrial applications and bioremediation.

## 2 MATERIALS AND METHODS

### 2.1 Materials

LAC (EC 1.10.3.2, a fungal LAC from Ganoderma lucidum) was purchased from Sigma‐Aldrich (St. Louis, MO). PU, CA (Mw = 131000 g/mol) was provided by Sinopharm Chemical Reagent (Shanghai, China). DMF, THF, 2,20‐azinobis‐(3‐ethylbenzothiazoline‐6‐sulfonic acid) (ABTS), FITC, coomassie brilliant blue (G‐250), 2‐methoxyphenol, 2‐bromoisobutyryl bromide (2‐BIB), 1,1,4,7,10,10‐hexamethyltriethyl‐enetetramine (HMTETA), triethylamine (TEA), HEMA, BPA were purchased from Aladdin Chemical Reagent (Shanghai, China) and used without further purification.

**PRACTICAL APPLICATION**

PU/RC‐poly(HEMA) nanofiber membrane could be used as a carrier for LAC immobilization and exhibited relatively higher stability and reusability. And it was further applied to waste water treatment with high removal efficiency on phenolic compounds.

### 2.2 Preparation of Fe(III)‐PU/RC‐poly(HEMA) blend nanofiber membrane

#### 2.2.1 Electrospinning

Prior to electrospinning, PU and CA mixture with mass ratio of 1/1 were dissolved in a mixture of DMF and THF solution at 45°C under the stirring condition. The electrospinning was carried out at 18 kV and the feed rate was set at 0.5 mL/h, meanwhile, the collector was placed at 17 cm below the tip of needle and the rotating speed of the collector was set at 100 rpm. The collected PU/CA membrane was hydrolyzed through immersion in 0.05 M NaOH aqueous solution at room temperature for 24 h. The resulting PU/RC nanofiber membrane was then rinsed in distilled water for three times and dried in a vacuum oven at 45°C.

#### 2.2.2 Initiation

PU/RC nanofiber membrane was first edulcorated by immersed in 20 mL of THF for 10 min, and then the nanofiber membrane was placed into a mixture of 70 µL of 10 mM TEA, 63 µL of 10 mM 2‐BIB and 50 mL of THF for 3 h at 35°C. 2‐BIB would react with hydroxyl groups on the surface of electrospun PU/RC nanofiber membrane, while TEA would neutralize the byproduct of HBr. After completion of the reaction, the nanofibers were taken out and stored in THF.

#### 2.2.3 Surface grafting

First, 24 mL of HEMA and a mixture of 400 µL of HMTETA and 24 mL of DMF were deoxygenated through three freeze‐pump‐thaw cycles by using liquid nitrogen, and then 100 mg of CuCl was added into the HMTETA/DMF mixture inside a vacuum glovebox followed by being magnetically stirred for 2 h. Subsequently, the initiated PU/RC nanofiber membrane and HEMA were placed into the mixture of HMTETA/DMF/CuCl, and the system was kept in the glovebox at 25°C for a predetermined time to complete the ATRP reaction of HEMA. Finally, the poly(HEMA)‐modified PU/RC nanofiber membrane was rinsed by ethanol and dried in air. According to the experimental results, the grafting rate of HEMA monomer in PU/RC‐poly(HEMA) nanofiber membrane was 23.1%.

#### 2.2.4 Ion coordination

Prior to immobilization, same weight of PU/RC and PU/RC‐poly(HEMA) nanofiber membrane was added to 40 mL of FeCl_3_ solution whose concentration was 100 mmol/L, the flack was shaken at 25°C for 24 h (120 r/min), then rinsed with deionized water. The obtained nanofiber membrane was dried in air and then dried at 35°C in a vacuum over couch.

### 2.3 Immobilization of LAC

**Figure 1:**
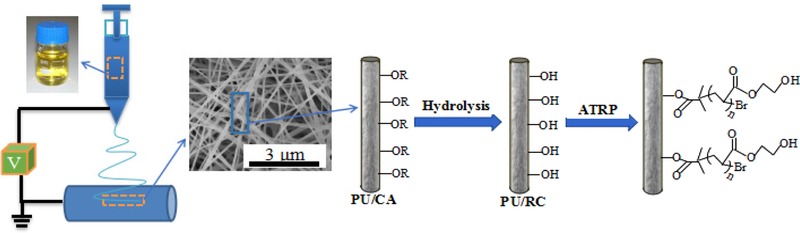
Schematic showing the grafting of poly(HEMA) on the surface of elecspun PU/RC nanofiber membrane via the ATRP method

**Figure 2:**
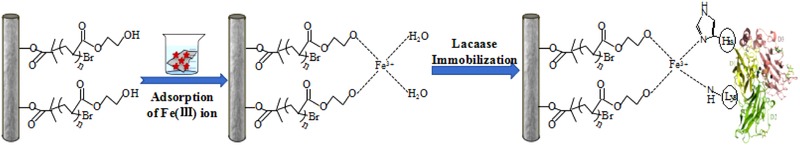
Schematic depicting the immobilization of LAC on PU/RC‐poly(HEMA) nanofiber membrane

For immobilization of LAC, the PU/RC, PU/RC‐poly(HEMA) nanofiber membrane was firstly immersed into 50 mL 3 g/L LAC solution (pH = 4) at 4°C for 12 h. Then, the membrane was removed from the solution and rinsed with HAc‐NaAc buffer solution until no soluble protein was detected, and the immobilized LAC was stored in HAc‐NaAc buffer solution at 4°C. The detailed procedure for making PU/RC‐poly(HEMA)‐LAC nanofiber membrane is schematically depicted in Figures 1 and 2.

In this experiment, the amount of immobilized LAC on the nanofiber membrane was determined by the method of Bradford. The fixed amount of LAC was determined by the Equation (1) 44:

(1) $$G_{\varepsilon }=\frac{\left(C_{0}-C_{1}\right)\times V_{0}-C_{2}\times V_{1}}{M_{d}},$$

Here, *G*_e_ is the amount of LAC bound onto unit mass of nanofibers (mg/g), *C*_0_ and *C*_1_ are the initial and equilibrium LAC concentrations in the solution (mg/mL), *V*_0_ is the volume of the LAC solution, *C*_2_ is the LAC concentration in the buffer solution used for washing the immobilized enzyme nanofiber, *V*_1_ is the volume of the buffer solution, and *M*_d_ is the mass (dry weight) of the nanofiber membrane.

### 2.4 Characterization of nanofiber membrane

Surface morphologies of the PU, PU/CA, PU/RC, PU/RC‐poly(HEMA), and PU/RC‐poly(HEMA)‐LAC membranes were examined by a Hitachi S4800 SEM. Image J software was used to determine the average nanofiber diameter, and 30 individual nanofibers in each SEM image were randomly selected for the diameter measurement. The presence of the fluorophore‐tagged LAC was determined using Confocal Laser Scanning Microscope technique, the LAC was labeled by FITC and the excitation and emission wavelengths were 488 nm. A Nicolet Nexus 470 FTIR spectrometer was employed to study the formation of different functional groups on the nanofibers, each type of membrane pieces was ground with potassium bromide (KBr) before being pressed into thin pellets for FTIR analysis, and the FTIR spectra were acquired by scanning the specimens for 32 times in the wavenumber range from 4000 to 450 cm^−1^ with the resolution of 4 cm^−1^. X‐ray photoelectron spectroscopy measurements were carried out on a Kratos XSAM800 XPS system with Kα source and a charge neutralizer. An Instron 1185 tensile strength tester was employed to test the mechanical properties of nanofiber membranes. The tensile rate and clamp distance were 10 mm/min and 2 cm with a membrane specimen sizexbrk of 5 × 1 cm.

### 2.5 Properties of free and immobilized LAC

#### 2.5.1 Activity assays

To acquire the respective activities of LAC under free and immobilized conditions, 0.1 mL of LAC solution (3 mg/mL, pH = 4), as well as nanofiber membrane with equivalent amount of immobilized LAC, were mixed with 2.9 mL ABTS solution (pH = 4). The system was first stored at 30°C for 3 min (five replicates for each membrane). The activities of free and immobilized LAC were determined spectrophotometrically at 420 nm. The catalytic activity of LAC was calculated by using the following Equation (2) 45:

(2) $$v=\frac{(A_{0}-A)\times V}{T\times K\times E_{W}},$$

where v is the specific activity of free or immobilized LAC (µ mol/mg·min); *A_0_* and *A* are the respective initial and final absorbance of solution at 420 nm; *V* is the volume of ABTS solution (mL); *T* is the reaction time (min); K is the molar extinction coefficient of ABTS at 420 nm; and *Ew* is the amount of LAC (mg).

In this experiment, relative activities were normalized to the initial activity which was taken as 100% by the following Equation (3):

(3) $$Rr=\frac{v}{v_{\max}}\times 100\%,$$

#### 2.5.2 Optimum reaction pH and temperature

To assess the pH dependence, the free and immobilized LAC were respectively incubated under different pH values (2.0–7.0) at 50°C for 5 min, subsequently, ABTS was added and the catalytic activities were then assayed. The highest catalytic activity of LAC was set as 100%, and then the relative catalytic activities under various conditions were then calculated.

In order to assess the temperature dependence, free and immobilized LAC were, separately, mixed with ABTS solution (pH = 4) in the temperature range from 30 to 70°C, immediately thereafter, the catalytic activities were assayed.

#### 2.5.3 Storage stability and reusability

Storage stability was evaluated upon calculating the residual activity of the free and immobilized LAC after being stored at 4°C in HAc‐NaAc for 24 days, and the highest value of each set was assigned as 100%. To examine the reusability of immobilized LAC, the activity of each nanofiber membrane with immobilized LAC was tested 10 times within 24 h. Prior to each test, the membrane with immobilized LAC was rinsed with HAc‐NaAc buffer solution to remove the reaction products and ABTS, and the activity of immobilized LAC was then tested in a fresh reaction medium.

### 2.6 BPA degradation

To evaluate the efficiency of LAC in the degradation of BPA, free and immobilized LAC were incubated with 100 µM BPA in a dark place for up to 2 h at 30°C and under agitation of 120 rpm. Degradation of BPA by free and immobilized LAC was determined according to the method of the Folin‐Ciocalteu spectrophotometric method. The reaction mixture (100 µL) was added to the mixture of Folin‐Ciocalteu reagent (500 µL) and deionized water (6 mL), and the mixture was shaken for 1 min. Then Na_2_CO_3_ solution (15 wt%, 2 mL) was added to the mixture and shaken for 1 min. Later the solution was brought up to 10 mL by adding deionized water. After 2 h, the absorbance was measured at 750 nm.

## 3 RESULTS AND DISCUSSION

### 3.1 Morphology analysis

**Figure 3:**
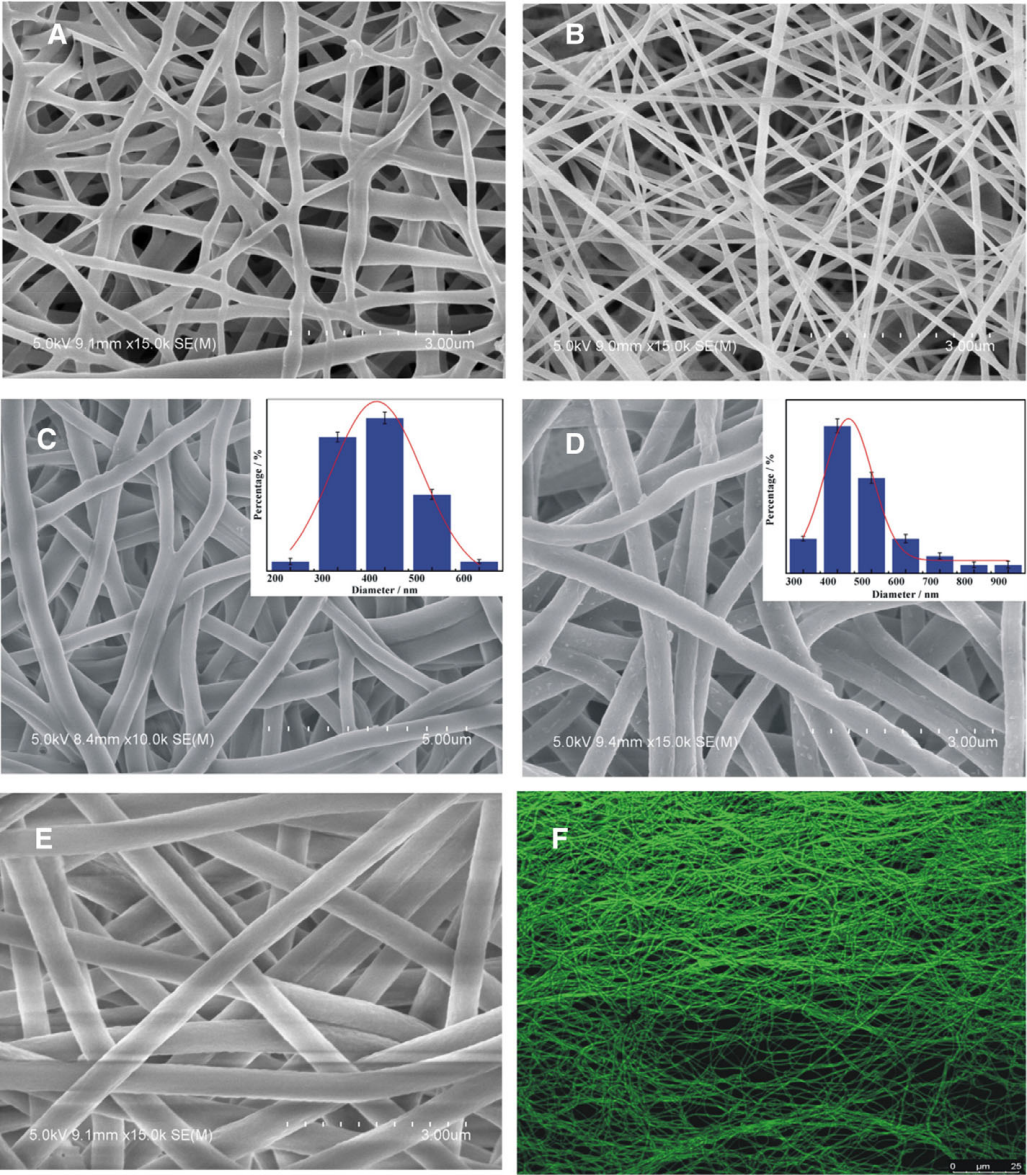
SEM images of different nanofiber membranes including (A) PU, (B) PU/CA, (C) PU/RC, (D) PU/RC‐poly(HEMA) (with inset showing the average diameter of PU/RC‐poly(HEMA)), (E) PU/RC‐poly(HEMA)‐LAC, and LSCM image of (F) PU/RC‐poly(HEMA)‐LAC

Figure 3 shows the LSCM image of PU/RC‐poly(HEMA)‐LAC and the SEM images of PU, PU/CA, PU/RC, PU/RC‐poly(HEMA), PU/RC‐poly(HEMA)‐LAC nanofiber membranes. An electrospun PU nanofiber membrane with unique network structure is shown in Figure 3A and Supporting Information Figure S1, because of the self‐bonding between the PU fibers due to the compatibility of its soft and hard segments while the organic solvent was not volatile in the process of electrospinning. The morphology of PU/CA nanofiber membrane (Figure 3B) was improved with the addition of CA, due to the phenomenon of self‐bonding was obviously reduced. Figure 3C shows the SEM image of PU/RC membrane, which average diameter with an obviously increase of 400 nm, due to the swelling produced by hydrolysis of CA. Followed, the obtained nanofiber membrane of PU/RC‐poly(HEMA) via ATRP modification, with a distinguishably addition in diameter to about 440 nm is shown in Figure 3D. Upon immobilization with LAC, the PU/RC‐poly(HEMA)‐LAC nanofiber (Figure 3E) with retained morphology and its diameter became considerably larger to about 540 nm, moreover, Figure 3F and Supporting Information Figure S2 show the 6‐aminofluorescein‐labelled LAC (green) was uniformly distributed on the backbone of the nanofiber. This analysis further confirms the large amount immobilized LAC on the membrane as calculated by the Bradford protein assay of 84.21 mg/g.

### 3.2 FTIR analysis

**Figure 4:**
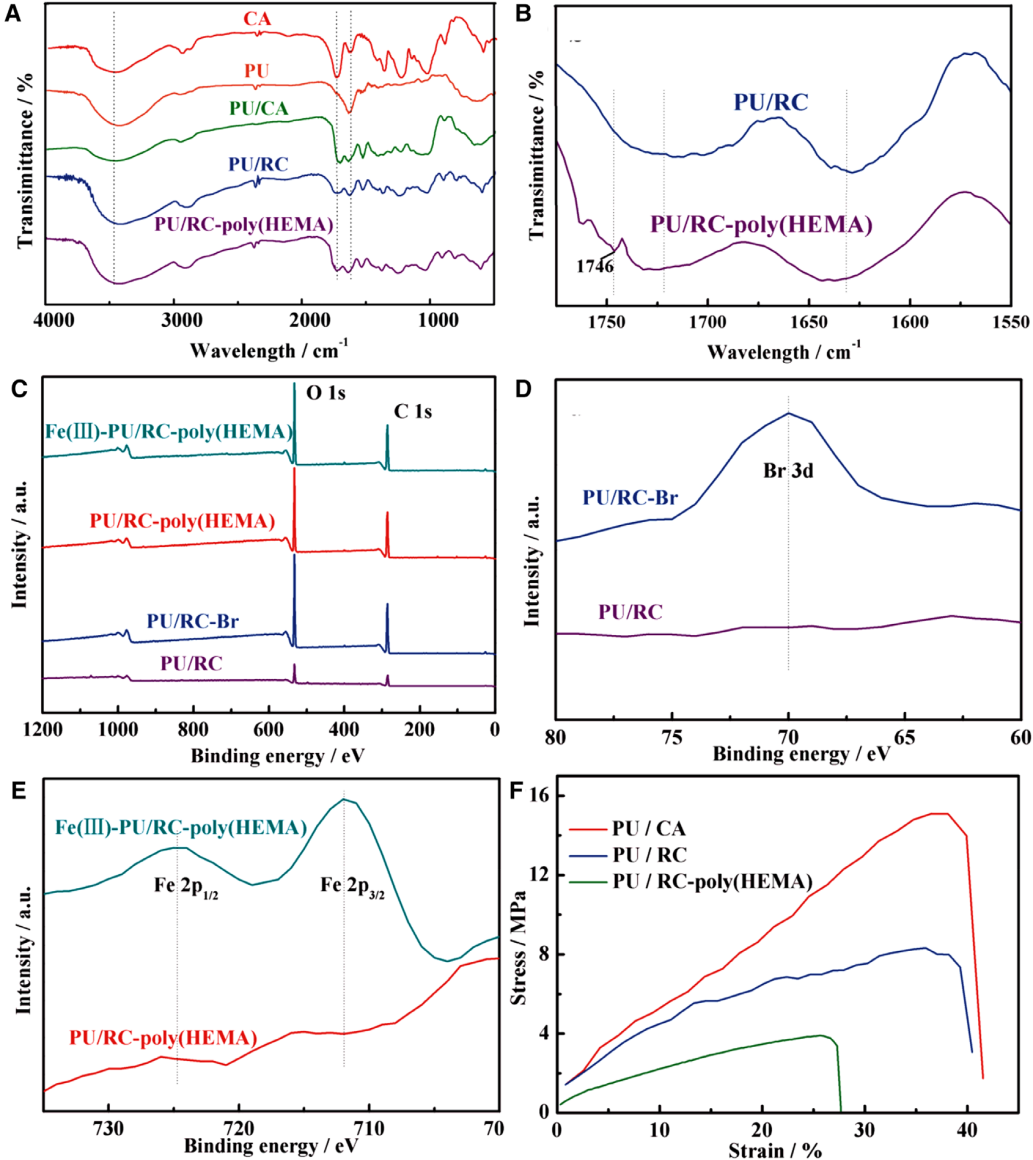
FT‐IR spectrum of nanofiber membranes (A) and magnified image at the range of 1775‐1550 cm^−1^ (B), XPS spectrum of nanofiber membranes (C) and partial enlarge in range of 80‐60 Ev (D) and 735‐700 eV (E), tensile stress‐strain curves acquired from nanofiber membrane

Figure 4A depicts FTIR spectra acquired from CA, PU, PU/CA, PU/RC, and PU/RC‐poly(HEMA) nanofiber membranes. As shown in Figure 4A, CA nanofiber membrane had three characteristic bands centered at the wavenumbers of 1726 cm^−1^ (C=O stretching), 1367 cm^−1^ (C–CH_3_ stretching) and 1231 cm^−1^ (C–O–C stretching); moreover, the two FTIR bands centered at 1630 and 1532 cm^−1^ in the spectrum of PU nanofiber membrane were attribute to the C=O, N–H stretching. All of the characteristic bands of PU and CA could be identified in the spectrum of PU/CA blend nanofiber membrane, indicating the co‐existence of both PU and CA in the nanofiber. After immersion in NaOH aqueous solution, CA was successfully converted into RC, as evidenced by the significant reduction of acetyl group related FT–IR bands centered at the wavenumbers of 1726 cm^−1^ (C=O), 1367 cm^−1^ (C–CH_3_), and 1231 cm^−1^ (C–O–C), in addition, the bands at 3500–3400 cm^−1^ can be respectively attributed to the stretching vibration of hydroxyl groups. The characteristic bands centered at 1746 cm^−1^ was obviously shown in Figure 4B, a magnified image at the wavenumber range from 1775 to 1550 cm^−1^ of Figure 4A, indicating the presence of ester groups were successfully surface‐grafted with HEMA via ATRP method.

### 3.3 X‐ray diffraction studies

Figure 4C shows the X‐ray photoelectron spectra (XPS) of PU/RC, PU/RC‐Br, PU/RC‐poly(HEMA) and Fe(Ⅲ)‐PU/RC‐poly(HEMA) nanofiber membranes. The survey spectrum indicates the presence of Br, Fe, C, and O elements, where the large peaks observed at 531.84 and 285.03 eV correspond to O 1s and C 1s, respectively. Two magnified images at the binding energy range from 80 to 60 eV and 735 to 700 eV are shown in Figure 4D and E, respectively, because the Br and Fe arrays on the surface of nanofiber membrane were ultrathin. The high‐resolution spectrum of Br 3d located at 70.02 eV (Figure 4D), confirms the binding of Br‐initiator to PU/RC nanofiber membrane. The peaks at 711.92 and 724.72 eV (Figure 4E) are related to the Fe 2p3/2 and Fe 2p1/2 binding energies of Fe^3+^ in Fe(Ⅲ)‐PU/RC‐poly(HEMA) nanofiber membrane, respectively. With compared to PU/RC‐poly(HEMA) membrane, indicated that iron ions were adsorbed on the surface of nanofiber membrane.

### 3.4 Mechanical properties

The stress‐strain curves of PU/CA, PU/RC and PU/RC‐poly(HEMA) nanofiber membranes were tested in this study and shown in Figure 4F. In specific, the ultimate stress and elongation at break of PU/CA membrane was 15.05 ± 0.27 MPa and (38.03 ± 0.16)%, superior to most nanofibers due to PU macromolecules contain carbonyl groups and amino groups, which can interact with some polar groupsthe. Followed, the stress and strain of PU/RC membrane remained at 8.28 ± 0.19 MPa and (35.79 ± 0.21)%, with a certain decline after hydrolysis modification. Thereafter, the membrane of PU/RC‐poly(HEMA) with a severe reduction in mechanical properties after ATRP modification, shown by the stress and strain being 3.93 ± 0.13 MPa and (25.78 ± 0.21)%, but still superior to those of most nanofibers. It is noteworthy that the mechanical properties acquired from PU/RC‐poly(HEMA) nanofiber membrane were substantially higher than the previously reported such as Neat PLA nanofiber membrane (3.4 ± 0.4 %) 46, CS/PVA nanafiber membrane (4.5 ± 1%) 47. It is necessary to note that the crucial contribution of PU component into blend nanofibers in improving the mechanical properties.

### 3.5 Optimum pH and temperature

**Figure 5:**
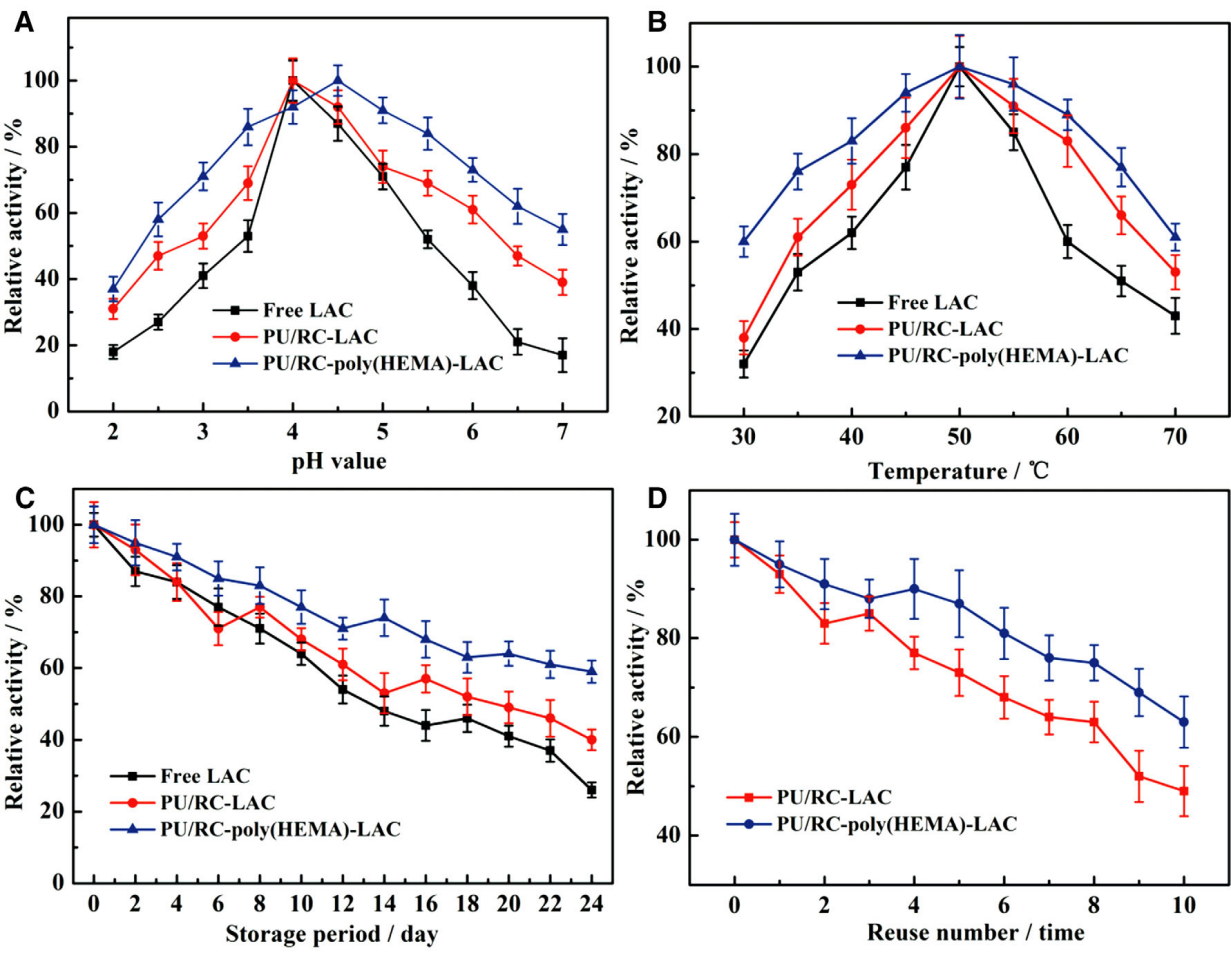
Effect of the reaction pH value (A) and temperature (B) on the catalytic activity of free and immobilized LAC, storage stability (C) of free and immobilized LAC and reusability (D) of immobilized LAC

A comparison study between the free LAC and the immobilized LAC of PU/RC‐LAC and PU/RC‐poly(HEMA)‐LAC was carried out to determine the optimum reaction temperature and pH value. As shown in Figure 5A, the optimal pH values with the highest enzyme activities were found to be 4.0, 4.0 and 4.5 for free, PU/RC‐LAC and PU/RC‐poly(HEMA)‐LAC, respectively. And it is evident that the catalytic activity of LAC is sensitive to pH value. Overall, PU/RC‐poly(HEMA)‐LAC showed a higher pH stability compared with free and PU/RC‐LAC system, a finding that can be attributed to the good affinity of PU/RC‐poly(HEMA) towards LAC. Therefore, PU/RC‐poly(HEMA)‐LAC exhibited the best tolerance over a broader range of pH value, a very useful finding for practical applications.

Figure 5B shows that the relationship between the relative activity of free and immobilized LAC with the temperature value in range from 30 to 70°C. It is very intuitive to find that, 50°C was the optimum temperature, both of free and immobilized LAC. Moreover, PU/RC‐poly(HEMA)‐LAC had higher relative activities than the free and PU/RC‐LAC in the entire temperature range from 30 to 70°C. The higher relative activity would primarily be attributed to the increased structural stability of PU/RC‐poly(HEMA)‐LAC molecules, while the multipoint interactions between LAC molecules and functional surface on the carriers might provide the further protection against inactivation at a higher temperature.

### 3.6 Storage stability and reusability

As shown in Figure 5C, the relative activity of free, PU/RC‐LAC, PU/RC‐poly(HEMA)‐LAC were (26 ± 2.1)%, (40 ± 2.9)%, and (59 ± 3.1)% after being stored at 4°C in HAc‐NaAc (100 mM, pH = 4) for 24 days, when the corresponding initial activities were set as 100%. The reduction of enzyme activity is a time‐dependent natural phenomenon; however, the degree of enzyme activity reduction could be mitigated considerably through immobilization. The immobilized enzyme molecules could better retain their conformational structure; therefore, the inactivation upon long‐term storage would be mitigated, thus ameliorating the storage stability of PU/RC‐poly(HEMA)‐LAC.

Unlike free LAC, the residual activities of PU/RC, PU/RC‐poly(HEMA) nanofiber membrane immobilized LAC remained (49 ± 5.1)% and (63 ± 5.2)% of their initial activities upon reuse 10 times (the nanofiber membrane with immobilized LAC were washed with HAc‐NaAc after each time) as shown in Figure 5D. It is well known that the reusability of an enzyme is among the major concerns for many practical applications. The results indicated that the PU/RC‐poly(HEMA)‐LAC nanofiber membrane exhibited better performance on reusability than PU/RC‐LAC under the same condition. For the PU/RC‐poly(HEMA)‐LAC nanofiber membrane, each repeat unit of poly(HEMA) brushes has a hydroxyl group, and the LAC molecules would be adsorbed/bound onto the PU/RC‐poly(HEMA) via hydrogen bonds and/or van der Waals forces. Thus, PU/RC‐poly(HEMA)‐LAC exhibited reasonably high performance on the reusability has a great value in the practical applications.

### 3.7 BPA degradation by free and immobilized LAC

**Figure 6:**
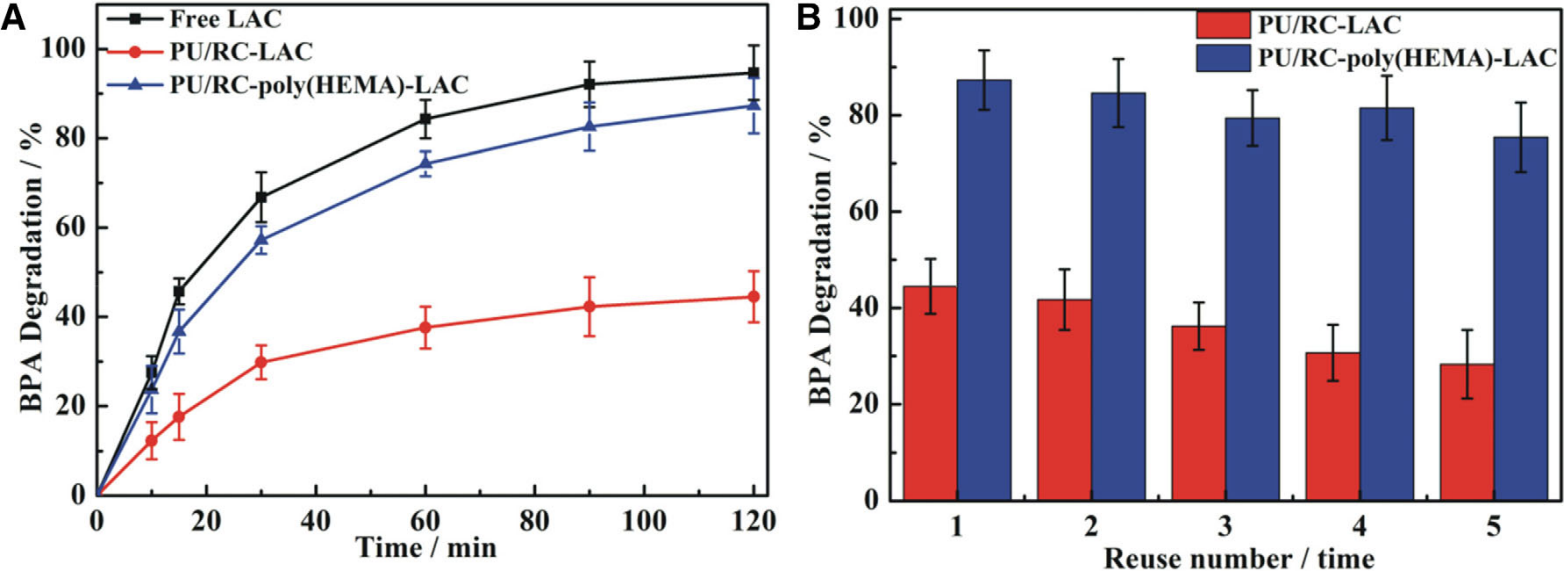
Removal efficiency of BPA by the free and immobilized LAC (A) and recyclability of the LAC‐modified membrane over five cycles (B)

The capability of free and immobilized LAC to remove the recalcitrant and hazardous pollutant BPA is shown and analyzed in Figure 6. Free LAC removed 94.7% BPA while PU/RC‐LAC and PU/RC‐poly(HEMA)‐LAC degraded 44.5 and 87.3% of BPA, respectively (Figure 6A) in a 2 h cycle study. It was indicated that the efficiency of the PU/RC‐poly(HEMA)‐LAC in removing BPA was far better than PU/RC‐LAC, and remained at levels similar to those predicted for the free LAC. In addition, according to the control experiments with no enzyme BPA adsorption on PU/RC and PU/RC‐poly(HEMA) membrane was no more than 10%. Therefore, LAC was mainly responsible for BPA degradation.

To evaluate the recyclability of the immobilized LAC, the removal of BPA (20 mg/L) was quantified during five successive batches of 2 h each. As shown in Figure 6B, the removal of BPA decreased gradually during the successive batches for both PU/RC‐LAC and PU/RC‐poly(HEMA)‐LAC. However, the removal of BPA by PU/RC‐poly(HEMA)‐LAC still maintained more than 75% capability, while PU/RC‐LAC decreased to lower than 30%. These results clearly showed that the LAC immobilized on modified membrane via ATRP method may be used with high efficiency in continuous reactors for phenolic removal in wastewater treatment.

## 4 CONCLUDING REMARKS

Herein, the electrospun PU/RC blend nanofiber membrane was surface‐grafted with HEMA via ATRP method and studied as innovative carrier for immobilization of LAC. The results acquired from LSCM and Bradford protein assay indicated that the LAC molecules were uniformly immobilized on the PU/RC‐poly(HEMA) nanofiber surface while the corresponding immobilization amounts were 84.21 mg/g, representing the improvements of about 300% as compared to the value of PU/RC nanofiber membrane (21.13 ± 4.3 mg/g). Moreover, the PU/RC‐poly(HEMA)‐LAC exhibited considerably improved resistance against the variations of temperature and pH value than the free and PU/RC‐LAC. Furthermore, the storage stability and reusability of the PU/RC‐poly(HEMA)‐LAC were significantly improved, while the residual activity was retained at (63 ± 5.2)% of its initial activity upon reuse 10 times within 1 day. Notably, the BPA removal efficiency of PU/RC‐poly(HEMA)‐LAC membrane generally ranged from 87.3 to 75.4% for the five cycles.

## CONFLICT OF INTEREST

The authors have declared no conflict of interest.

## Supporting information

Supporting Information
