# Supplementary material for: Immobilization of laccase onto modified PU/RC nanofiber via atom transfer radical polymerization method and application in removal of bisphenol A
Source: Eng Life Sci. 2019 Nov 4;19(11):815–24. doi: 10.1002/elsc.201900075 (PMC6999588; doi:10.1002/elsc.201900075)
Supplement: Supplementary file 1 — Supporting Information [file ELSC-19-815-s001.pdf]

Supporting Information

of

Immobilization of Laccase onto Modified PU/RC Nanofiber via Atom Transfer Radical Polymerization Method and Application in Removal of Bisphenol A

Xin Li[1], Dawei Li[1], Pengfei Lv[1], Jinyan Hu[3], Quan Feng*[,3], Qufu Wei*[,1,2]

[1] Key Laboratory of Eco-Textiles, Ministry of Education, Jiangnan University, 1800 Lihu Avenue, Jiangsu Province, Wuxi 214122, PR China

[2] Fujian Key Laboratory of Novel Functional Textile Fiber and Materials, Minjiang University, Fuzhou, Fujian, 350108, PR China

[3] Key Laboratory of Textile Fabric, Anhui Polytechnic University, Wuhu, Anhui 241000, PR China

*Corresponding authors. Email addresses: fengquan@ahpu.edu.cn(Q. Feng); qfwei@jiangnan.edu.cn (Q. Wei)

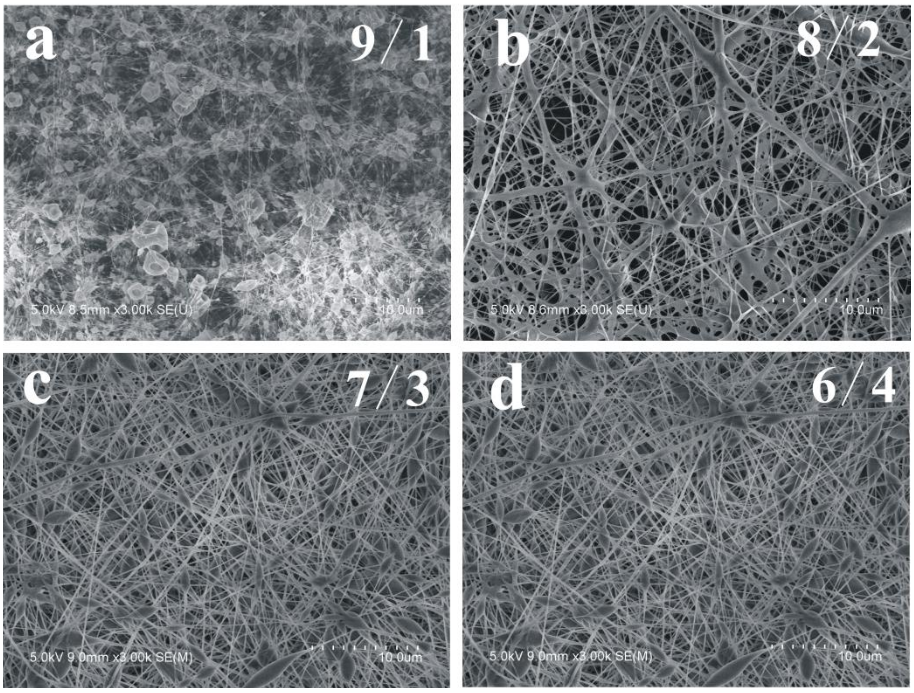

Fig. S1 SEM images of PU/CA nanofiber membranes with the mass ratio of CA and PU at (a) 9/1,

(b) 8/2, (c) 7/3 and (d) 6/4.

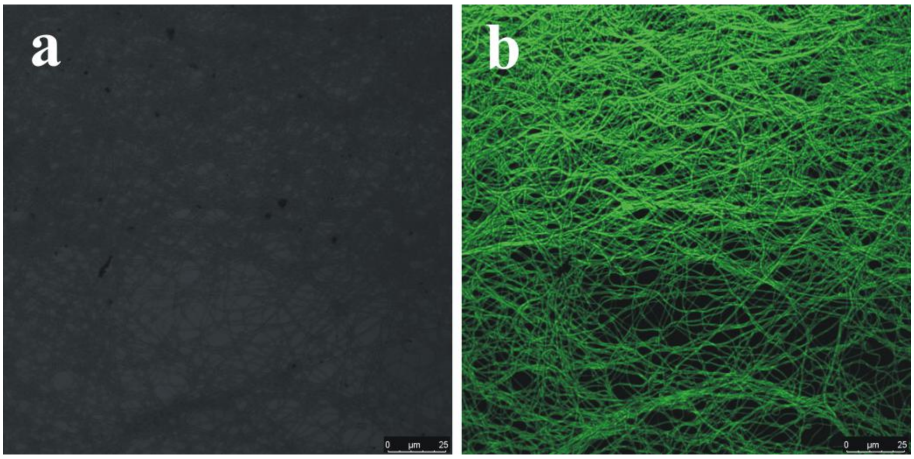

Fig. S2 CLSM showing the (a) bright field and (b) fluorescence images of PU/RC-poly(HEMA)-LAC

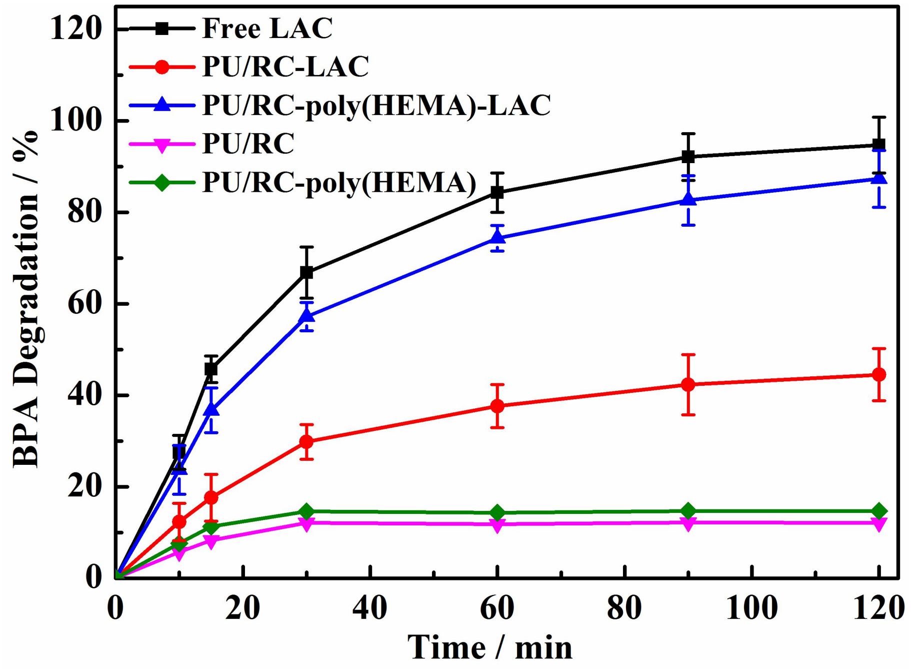

Fig. S3 Removal efficiency of BPA by different samples.

References:

1. Quan, F.; Yong, Z.; Anfang, W.; Changlong, L.; Qufu, W.; Hao, F. Environmental Science & Technology 2014, 48 (17), 10390.

2. Bilal, M.; Asgher, M.; Shahid, M.; Bhatti, H. N. International Journal of Biological Macromolecules 2016, 86, 728-740.

3. Tavares, A. P. M.; Silva, C. G.; Dražić, G.; Silva, A. M. T.; Loureiro, J. M.; Faria, J. L. J Colloid Interface Sci 2015, 454, 52-60.

4. Mohajershojaei, K.; Mahmoodi, N. M.; Khosravi, A. Biotechnology & Bioprocess Engineering 2015, 20 (1), 109-116.
